# The monoclonal antibody 17E1 selectively labels GFAP in 5xFAD mice

**DOI:** 10.1002/alz.095637

**Published:** 2025-01-09

**Authors:** Christopher M. Norris, Susan D Kraner

**Affiliations:** ^1^ University of Kentucky College of Medicine, Sanders‐Brown Center on Aging, Lexington, KY USA

## Abstract

**Background:**

Our lab recently developed 2 mouse monoclonal antibodies that preferentially react with “distressed astrocytes”. One monoclonal, 26A6, was found to react preferentially with a form of the Ca2+/calmodulin‐dependent protein phosphatase, calcineurin (CN), that has been cleaved by calpain, to generate a 48 kDa CN fragment (∆CN). We recently published a characterization of this antibody. Surprisingly, the second antibody (17E1), generated to the same antigen used to create 26A6, preferentially binds to GFAP and is present at elevated levels in the 5xFAD mouse strain.

**Method:**

A peptide encompassing the calpain sensitive region of the CN carboxyl terminus was used for mouse monoclonal antibody generation.

**Result:**

We identified 2 monoclonal antibodies: 26A6 and 17E1. The 26A6 antibody binds to a recombinant CN fragment (48 kDa) that is generated by calpain‐mediated proteolysis. We’ve shown that this antibody selectively labels subsets of reactive astrocytes in human brain in conjunction with Alzheimer’s and cerebrovascular pathology, but provides very little labeling in healthy control brain tissue. The 17E1 antibody also labels a 48 kDa protein, but does not label recombinant CN, nor does it label calpain generated CN fragments. Western blot analyses found that the 17E1 and 26A6 antibodies exhibit differential labeling of a 48 kDa protein found in 5xFAD mice. Specifically, 26A6 showed no labeling while 17E1 exhibited highly selective labeling. The 48 kDa band was also detected with a commonly used commercial CN antibody from EMD Millipore (1492) that labels full length CN and multiple C terminus truncation products of CN. To our surprise, immunoprecipitation experiments followed by Western and mass spectroscopy showed that the 48 kDa fragment bound by both 17E1 and 1492 was actually GFAP. Both the 17E1 and 1492 Abs label recombinant GFAP, but 26A6 did not. 26A6 also exhibited no substantial labeling in 5xFAD brain tissue.

**Conclusion:**

Both 26A6 and 17E1 preferentially interact with “distressed astrocytes”. However, 26A6 labels proteolyzed CN, while 17E1 labels GFAP. The results emphasize the importance of using the correct antibodies to evaluate the role of CN in neurodegenerative disease. Further characterization of 17E1 in human brain tissue and rodent models of disease is underway.

